# Microarray-Based Analysis of the Differential Expression of Melanin Synthesis Genes in Dark and Light-Muzzle Korean Cattle

**DOI:** 10.1371/journal.pone.0096453

**Published:** 2014-05-08

**Authors:** Sang Hwan Kim, Sue Yun Hwang, Jong Taek Yoon

**Affiliations:** 1 Institute of Genetic Engineering, Hankyong National University, Ansung, Kyeonggido, Korea; 2 Department of Chemical Engineering, Hankyong National University, Ansung, Kyeonggi-do, Korea; 3 Department of Animal Life Science, Hankyong National University, Ansung, Kyeonggido, Korea; University of Lausanne, Switzerland

## Abstract

The coat color of mammals is determined by the melanogenesis pathway, which is responsible for maintaining the balance between black-brown eumelanin and yellow-reddish pheomelanin. It is also believed that the color of the bovine muzzle is regulated in a similar manner; however, the molecular mechanism underlying pigment deposition in the dark-muzzle has yet to be elucidated. The aim of the present study was to identify melanogenesis-associated genes that are differentially expressed in the dark vs. light muzzle of native Korean cows. Using microarray clustering and real-time polymerase chain reaction techniques, we observed that the expression of genes involved in the mitogen-activated protein kinase (MAPK) and Wnt signaling pathways is distinctively regulated in the dark and light muzzle tissues. Differential expression of tyrosinase was also noticed, although the difference was not as distinct as those of MAPK and Wnt. We hypothesize that emphasis on the MAPK pathway in the dark-muzzle induces eumelanin synthesis through the activation of cAMP response element-binding protein and tyrosinase, while activation of Wnt signaling counteracts this process and raises the amount of pheomelanin in the light-muzzle. We also found 2 novel genes (GenBank No. NM-001076026 and XM-588439) with increase expression in the black nose, which may provide additional information about the mechanism of nose pigmentation. Regarding the increasing interest in the genetic diversity of cattle stocks, genes we identified for differential expression in the dark vs. light muzzle may serve as novel markers for genetic diversity among cows based on the muzzle color phenotype.

## Introduction

According to their coat color, native Korean cows (HanWoo) are classified as brown, black, white, brindle, and mixed. Due to the increasing interest in the genetic diversity of cattle stocks, in 2004, the Korean Ministry of Agriculture published a National Report on the current status of the gene pool among cattle, where native Korean cows are categorized as Yellow HanWoo, Black HanWoo, Jeju Black HanWoo, and Brindle HanWoo. This report has promoted efforts not only to preserve the genetic diversity prevalent among cows but also to investigate the genes associated with the expression of the color of bovine coats. The coat and muzzled colors in mammals are determined by the relative distribution of pheomelanin and eumelanin. In melanocytes, the expression of these two pigments is controlled by the melanocortin 1 receptor (MC1R) as well as by agouti locus alleles [1], [2], [3]. To date, single-nucleotide polymorphisms (SNPs) associated with the expression of coat color have been identified in the *MC1R* gene [4], [5], [6], [7], as well as in other genes associated with pigment deposition, such as tyrosinase (TYR), tyrosinase-related protein (TYRP-1), dopachrome tautomerase (DCT), and *agouti*(ASIP) [3], [8], [9]. Among these, the role of TYR is of note in that generally melanin is synthesized from tyrosine through dopa and dopa-quinone, a process controlled by TYR [8], [9].

In contrast to skin color, limited information is available on the nature of pigment deposition in the nose, although this could also be a marker reflecting genomic diversity. According to the system developed by Lee et al. (2002), the degree of darkness of the cow's muzzle is determined by the number of black spots in the muzzle. Interestingly, it has been shown that genetic variation in the MC1R locus is also associated with the color of the muzzle. Both E+/e and e/e genotypes are prevalent among Yellow HanWoo, but the frequency of the E+ allele is higher in animals with dark-muzzle than in the general population (0.37 vs. 0.11, respectively) [10], [11], [12]. However, little information is available on genes other than *MC1R* that control pigment expression in the cow's muzzle.

In an attempt to gain further information about the genes controlling pigmentation of the muzzle, we compared the gene expression profile of the muzzle tissue between native Korean cows with and without black spots in their muzzle. We found an intriguing distinction between the dark and light muzzle with respect to the expression of genes involved in the MAPK vs. Wnt signaling pathways. Our results provide new insights into the molecular mechanism of melanogenesis in the cow's muzzle.

## Materials and Methods

### Animals and Tissues

Cows examined in this study were all raised under the same condition at a farm affiliated to Hankyong National University. Animals were mostrly kept indoors except occasional outdoor exercise period.

**Figure 1:**
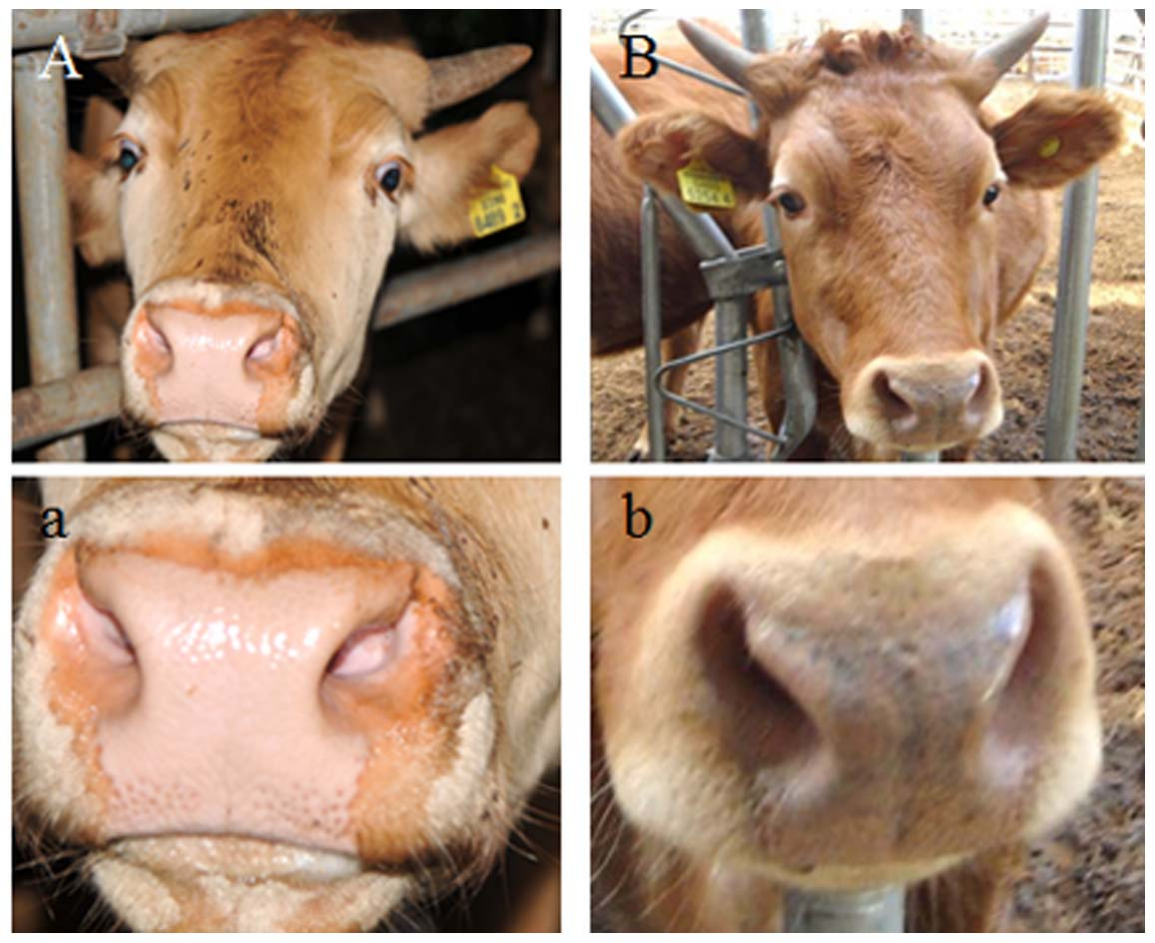
Criteria for the classification of the muzzle and coat color of HanWoo. A–B) Yellow coat color, a: Light-muzzle, b: Dark-muzzle.

The muzzle tissue of cows was obtained from the Pyong-Nong slaughter house in Pyungtaeck, Gyeonggi-Do, Korea (obtained permission No: HK20130103-010). Pigment deposition in the muzzle was categorized as either strong black, medium black, weak black, and yellow, according to the system developed by Lee et al. (2012). Only cows of verified pedigree, as ascertained by the official number issued by the Rural Development Administration, were used for this study, and 18 medium-dark-muzzled and 18 light-muzzled animals were selected for tissue preparation (Fig. 1). The front part of the muzzle was surgically removed and was directly placed in liquid nitrogen (−196°C). This study was carried out in strict accordance with the recommendations in the Guide for the Care and Use of Laboratory Animals of the National Institutes of Health. The protocol was approved by the Committee on the Ethics of Animal Experiments of the Hankyong National University (Permit Number:2013-1).

### Microarray

Total RNA was extracted from the muzzle tissues of one representative yellow-se cow and one representative black-nose cow using Trizol reagent (Invitrogen, CA, USA) according to the manufacturer's procedure. The concentrations of RNA present in the samples as well as the OD 260/280 ratio of the samples were determined using a nano-drop spectrophotometer. Reverse transcription of the first strand of complementary DNA (cDNA) was performed using an oligo dT primer (Invitrogen, CA USA). Bovine glyceraldehyde-3-phosphate dehydrogenase (GAPDH) was used as an internal control for quantitation.

The NCBI Gene Expression Omnibus (GEO) accession number for the microarray data reported in this paper is GSE53657 and the data are available at http://www.ncbi.nlm.nih.gov/geo/. Also available for download from this GEO accession is a *Supplementary Analysis File* containing all pre-processing analyses, annotated lists of differentially expressed genes with links to NCBI as well as gene ontology and pathway analyses (http://www.ncbi.nlm.nih.gov/geo/query/acc.cgi?acc=GSE53657).

Microarray-based analyses of the gene expression profile in the tissue samples were performed by ISTECH Inc. (http://www.istech21.com, Seoul, Korea) using the bovine 4*44K chip (Chip no. 252364710266, Agilent Technologies Inc., CA USA). Briefly, purified RNAs were tested for their quality with the Agilent 4-plex gasket System (Agilent Technologies Inc., CA USA), followed by amplification and labeling using Agilent's low RNA input linear amplification kit PLUS (Agilent Technologies Inc., CA, USA). Microarray hybridization was carried out using the Agilent Gene Expression Hybridization kit, washed using a series of buffers from the Agilent Gene Expression Wash Buffer Kit, and then scanned and analyzed using an Agilent DNA microarray scanner and Feature Extraction Software, respectively. The images obtained were normalized and clustered using Agilent Gene Spring Software (Agilent Technologies Inc., CA, USA). And cluster analysis of the molecular characteristic and biological information of genes in the cattle was using KEGG PATHWAY database (http://www.genome.jp/kegg, JPN).

### Real-time polymerase chain reaction

The specificity of genes showing significant differences in expression between the 18 dark-muzzled and 18 light-muzzled cattle were confirmed using real-time polymerase chain reaction (RT-PCR). Cows examined by the microarray analysis were included among the 18 cows from each group. Primers for amplification of genes of interest were prepared as listed in Table 1. RNAs were prepared using the same method as described for the microarray analyses. Reverse transcription and PCR amplification were performed using the one-step SYBR RT-PCR kit (TaKaRa, Shiga, Japan) and line-gene K program (Bioeer Technology, Tokyo JPN). Each PCR was repeated at least 3 times, and the results were converted as a fold increase according to the cycle threshold (Ct) values in the semi-log amplification plot of the geometric region, using Rotor-Gene Real-Time Software 6.0 (BIOER, Tokyo Japan).

**Table 1:** Primers for real-time polymerase chain reaction analysis of melanogenesis-associated genes.

Primer		Sequence	Product Size	Primer		Sequence	Product Size
MAGEB3	Fw	*5′ CAGACCGCACATTGGAGAGAGG 3′*	*229*	CREB3L4	Fw	*5′ CAGTCAGCTCAGGATAGCCG 3′*	*208*
	Rv	*5′ GCCACCACGATGGAAGCACTC 3′*			Rv	*5′ CACAAGTGCTAGTCTGGGCA 3′*	
CALML5	Fw	*5′ GAAGGTGTCTCAGGACGAGC3′*	*203*	FGF11	Fw	*5′ GTCGCTTTAAGGAGTGCGTC 3′*	*213*
	Rv	*5′ CCAACTGTCCCCACATGGAA3′*			Fw	*5′ CACTGTGGAGAGAAGGCTCC 3′*	
CAMK2A	Fw	*5′ ATCGCCTATATCCGCATCAC 3′*	*206*	FGF7	Rv	*5′ GTGAGAAGACTGTTCTGTCG 3′*	*380*
	Rv	*5′ GGACAAAGAGCGGATCTCTG 3′*			Fw	*5′ GCCATAGGAAGAAAATGGGC 3′*	
CAMK2B	Fw	*5′* ATGGCACATGAGGCACTGAA *3′*	*176*	FZD1	Fw	*5′ CGAGCTGGGAACTTTGTGC 3′*	*242*
	Rv	*5′ GTCTGTCTGCCCACTGAGAA 3′*			Rv	*5′ GTACTGCTGCCCGCTCTGTT 3′*	
PRKACB	Fw	*5′ GCTTCCCTTTGGTTCGTCATTC 3′*	*205*	FZD8	Fw	*5′ CTGGATTTGCCTCCGTGAGT3′*	*213*
	Rv	*5′ CCTCAAGCAACTGACCAACACA 3′*			Rv	*5′ CGAGCAAGTTTCTTCTCGG3′*	
PDGFRB	Fw	*5′ AATCCTTGTGATGCCAGGGG 3′*	*111*	FZD9	Fw	*5′ CAATACGGAGAAGCTGGAG 3′*	*255*
	Rv	*5′ ATCCTTGCTGCTGATGCCTT 3′*			Fw	*5′ CCCACCACCAAGGACATGAA3′*	
FGF13	Fw	*5′ CTGTGGAACTGTGATGTTG 3′*	*141*	HSPA5	Rv	*5′ GAAAATGCTATAGCCCAAGTGG 3′*	*141*
	Rv	*5′ TGTTGTTTAGGGGTAACCAGTC 3′*			Fw	*5′ CAAGGTGAACACACACCCTG3′*	
FGF16	Fw	*5′ CTCCTTGGACTGGGACCTGC 3′*	*530*	MAGEB5	Fw	*5′ GCCTCGGAAGCACAAAAACA 3′*	*184*
	Rv	*5′ AGTGAGTGAATTTCTGGTGTCG 3′*			Rv	*5′ CTGAAGCTCCTGGTGATCGG 3′*	
FGF10	Fw	*5′ ATCACCTCCAAGGAGATGTCCG 3′*	*156*	MAGEE2	Fw	*5′* TGGATCGGGTCTTTGGGTTG*3′*	*123*
	Rv	*5′ CGGCAACAACTCCGATTTCCAC 3′*			Fw	*5′ CCTTTGGCATGTCCAGGGAT 3′*	
FGF4	Fw	*5′ TACTGCAACGTGGGCATCGGA 3′*	*346*	MAGEF1	Rv	*5′ ACCTGCCTACCCGAAAACAT3′*	*868*
	Rv	*5′ GTGGGTTACCTTCATGGTAGG 3′*			Fw	*5′ CCTCACGGTACTGCACTGG3′*	
ADCY2	Fw	*5′ GAAGACCACGTGGCATTTTT 3′*	*166*	MAGEH1	Fw	*5′ TTATAAGCCGGTGCCCCGT3′*	*198*
	Rv	*5′ CGAAACACATGAACAAGTAGCC 3′*			Rv	*5′ GCACCTGAATTGAGGATAGC 3′*	
TYR	Fw	*5′ TAACAGAACCTGCCAGTGC 3′*	*90*	MIA	Fw	*5 ′TAGCATCGTACGTGAAGACCA 3′*	*117*
	Rv	*5′ CTTTCTGTGCAGCGGGGTCCCCTAGA 3′*			Rv	*5′ GGGAGAAACAACAGCAATGACA 3′*	
ADCY6	Fw	*5′ AGGTTCTAGCGGCCAAGG 3′*	*800*	MLPH	Fw	*5′ CCAGCCCTAAGAGGAGAGGT 3′*	*195*
	Rv	*5′ TAATGCCCTCATGCACATTC 3′*			Fw	*5′ ACTTGGGTGAAGAGCTGACG 3′*	
CCND1	Fw	*5′ CGATGCCAACCTCCTCAACGAC 3′*	*143*	P4HB	Rv	*5′ GAGGGAGCGTCGATTGGAAATG 3′*	*329*
	Rv	*5′ CCAGCATCCAGGTGGCGACG 3′*			Fw	*5′ AGCACACACCCTGACGTCCAAA 3′*	
CREB3L2	Fw	*5′ GCAAAGTGGCCAAACCTGACTA 3′*	*269*	PRAME	Fw	*5′ AGCAGCTTCTGTGCAAGTCT 3′*	*102*
	Rv	*5′ TGAACTCAGAGAAGACGGAGCA 3′*			Rv	*5′ GGATGGGCTCGGTGTCATAG 3′*	

### *In situ* hybridization

Probes specific to the highly expressed genes in dark-muzzle cows were generated by PCR amplification and labeled using a digoxigenin-labeled hybridization kit (Roshe, Mannheim, Germany), according to the manufacturer's procedure. Tissues for hybridization were prepared from the epithelium and dermis containing melanocytes and fixed for 24 h in 70% ethanol (EtOH)/0.2% diethyl pyrocarbonate (DEPC). Following a series of dehydration steps, the tissues were embedded in paraffin blocks and mounted as 10-µm sections. After deparaffinization, the sections were hybridized with the labeled probes by incubating in RiboHybe hybridization solution (TOYOBO, Osaka, Japan) for 16 h at 65°C. The hybridized sections were then re-fixed in 0.2×SSC containing 60% formamide. Color development was induced by binding anti-digoxigenin antibody for 2 h at 37°C, followed by incubation for 2 h with NBT/BCIP stock solution (0.18 mg/ml BCIP, 0.34 mg/ml NBT, and 240 µg/ml levamisole) in the dark. The sections were then treated with methyl green to stain the nucleus followed by observation under a light microscope.

### Statistical Analyses

The Microarray and real-time RT-PCR results were analyzed for statistical significance using the SAS package (Statistical Analysis System, Institute, version 9.4, Cary, NC, USA). Data were subjected to a Welch's T-test, fold change and GLM of the SAS. The data are shown as mean ± SD, and the significant difference between groups was determined at the *p*<0.05 level.

## Results

### Microarray comparisons of gene expression profiles between cows with dark vs. light muzzle

**Figure 2:**
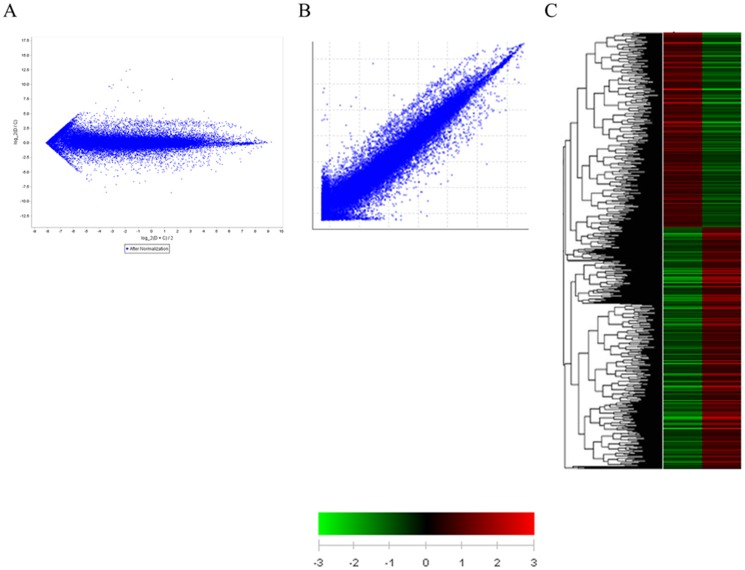
Visualization of clustered microarray data. A) MA plot, B) Scatter plot, and C) Hierarchical clustering analyses of 5,094 selected genes. The red color indicates the up-regulation of gene expression in the dark-muzzle HanWoo compared to that of the light-muzzle HanWoo. The green color indicates the down-regulation of genes.

Using the Agilent bovine 4*44K chip containing 44,000 cow genes, we compared the gene expression profiles of the muzzle tissue from dark and light muzzle cows. MA plots and scatter plots were applied for normalization and clustering to minimize possible variations and errors (Fig. 2A and B). In this test, 2,820 genes showed increased expression in the tissue of cows with a dark-muzzle as compared to that of light-muzzle cows, whereas 2,274 genes showed decreased expression. These 5,094 genes were further analyzed for the significance of differential expression through hierarchical clustering. As a result, up-regulated genes with significantly higher expression in dark-muzzle cows (red color) and down-regulated genes with significantly decreased expression (green color) were identified (Fig. 2C).

### Identification of genes associated with Melanogenesis

From the results of gene clustering, we identified genes associated with melanogenesis that were differentially expressed in tissues of the dark and light-muzzle cows. Among these, 12 showed a more than 2-fold increase in expression in dark-muzzle cows, whereas the expression of 18 genes was significantly decreased (Tables 2, 3).

**Table 2:** List of melanogenesis and MAPK signaling associated genes that are up-regulated in the dark-muzzle tissue.

Genes	Description	Genbank ID	Fold Increase
MAGEB3	*Bos taurus* MAGE-B3-like	XM_002700267	3.43
CALML5	*Bos taurus* calmodulin-like 5	NM_001098049	3.47
CAMK2A	*Bos taurus* calcium/calmodulin-dependent protein kinase II alpha	NM_001075938	12.84
CAMK2B	*Bos taurus* calcium/calmodulin-dependent protein kinase II beta	NM_001035357	7.04
PRKACB	*Bos taurus* protein kinase, cAMP-dependent, catalytic, beta	BC149047	2.10
PDGFRB	*Bos taurus* platelet-derived growth factor receptor, beta polypeptide	NM_001075896	2.05
FGF13	*Bos taurus* fibroblast growth factor 13	NM_001098892	5.51
FGF16	*Bos taurus* fibroblast growth factor 16	NM_001192777	2.53
FGF10	*Bos taurus* fibroblast growth factor-10	AY183659	2.50
FGF4	*Bos taurus* fibroblast growth factor 4	NM_001040605	2.02
ADCY2	*Bos taurus* adenylate cyclase 2	XM_587884	4.82
TYR	*Bos taurus* tyrosinase	NM_181001	3.10

**Table 3:** List of melanomagenesis and MAPK signaling associated genes that are down-regulated in the dark-muzzle tissue.

Genes	Description	Genbank ID	Fold Decrease
ADCY6	*Bos taurus* adenylate cyclase 6	NM_001143877	2.48
CCND1	*Bos taurus* cyclin D1	NM_001046273	1.09
CREB3L2	*Bos taurus* cAMP responsive element binding protein 3-like 2	NM_001102533	2.50
CREB3L4	*Bos taurus* cAMP responsive element binding protein 3-like 4-like	XM_880784	2.10
FGF11	*Bos taurus* fibroblast growth factor 11	NM_001192939	1.09
FGF7	*Bos taurus* fibroblast growth factor 7	NM_001193131	2.49
FZD1	*Bos taurus* frizzled homolog 1	NM_001101048	1.05
FZD8	*Bos taurus* frizzled-8-like	XM_869051	2.48
FZD9	*Bos taurus* frizzled homolog 9	XM_599625	2.07
HSPA5	*Bos taurus* heat shock 70kDa protein 5	NM_001075148	1.41
MAGEB5	*Bos taurus* misc_RNA	XR_083430	1.16
MAGEE2	*Bos taurus* melanoma antigen family E, 2	NM_001076876	2.45
MAGEF1	*Bos taurus* melanoma antigen family F, 1	NM_001102049	2.47
MAGEH1	*Bos taurus* melanoma antigen family H, 1	NM_001080728	2.38
MIA	*Bos taurus* melanoma inhibitory activity	NM_173936	1.50
MLPH	*Bos taurus* melanophilin	NM_001081597	2.22
P4HB	*Bos taurus* prolyl 4-hydroxylase, beta polypeptide	NM_174135	1.22
PRAME	*Bos taurus* preferentially expressed antigen in melanoma transcript 1	GU144301	2.26

In particular, the *PRKACB* (cAMP-dependent, catalytic, beta) gene, which controls cAMP responsive element binding protein 1 expression (CREB), *TYR*, and calcium/calmodulin-dependent protein kinase II alpha (*CAMK2α*) exhibited a more than 12-fold increase in expression in dark-muzzle cows compared with light-muzzle cows. On the other hand, expression of the frizzled family gene (*FZD*), which is the upstream regulator of the Wnt signaling pathway, was weaker in dark-muzzle cows than in light-muzzle cows.

### Verification of differential expression by real-time RT-PCR

To confirm the results of microarray analyses, 30 genes whose expression varied significantly between the dark and light muzzle cows, were tested using real-time RT-PCR. For this purpose, we collected muzzle tissue samples from 18 dark-muzzle and 18 light-muzzle cows.

**Figure 3:**
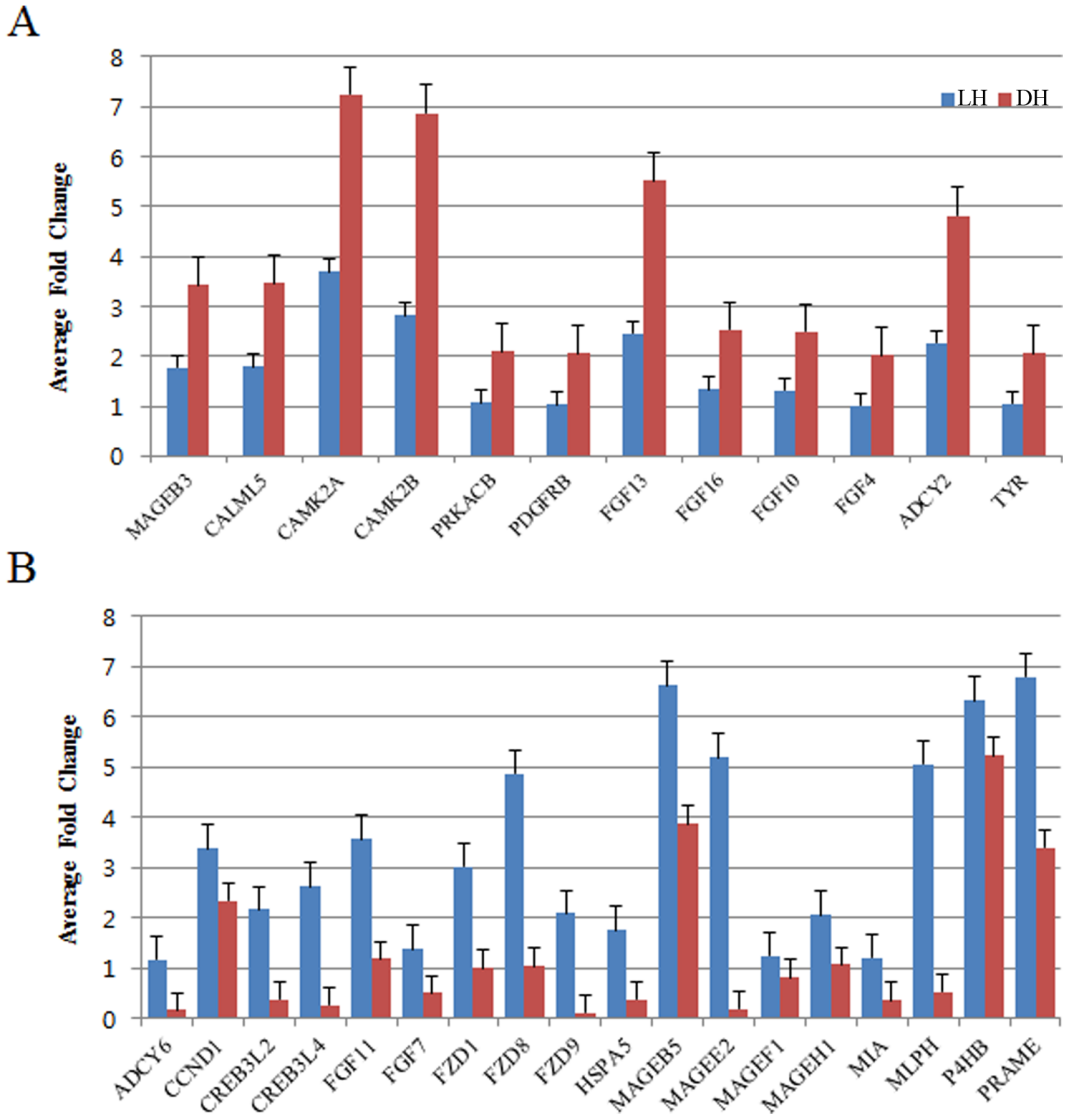
Real-time RT-PCR analysis of melanogenesis-associated genes between the muzzle tissues of dark-muzzle HanWoo (DH) and light-muzzle HanWoo (LH). The bars represent the average fold changes from 3 independent experiments (±SD). A) Genes involved in the upstream pathways of melanogenesis. B) Genes involved in the downstream pathways of melanogenesis.

As shown in Fig. 3, RT-PCR of these 30 genes revealed expression patterns similar to those obtained using the microarray. In particular, higher expression was confirmed in dark-muzzle cows for *ADCY2*, which activates microphthalmia-associated transcription factor (MITF) downstream to MC1R, as well as genes that are associated with PRKACB and MAPK signaling, such as *PDFRB* and *FGF4*, *10*, *13*, and *16*; and factors directly involved in melanogenesis such as *TYR*, *CAML5*, and *CAMK2α/β*. On the other hand, increased expression of MITF-activating factors such as *ADCY6*, *CCND1*, *CREB3L2/4*, and *FGF11* and *7* in the MAPK signaling pathway and *FZD1*, *8*, and *9* associated with the Wnt signaling pathway. In addition, several genes known as the melanoma antigens were also highly expressed in tissues from light-muzzle cows.

### Expression analyses of novel genes with strong expression in the dark-muzzle

**Figure 4:**
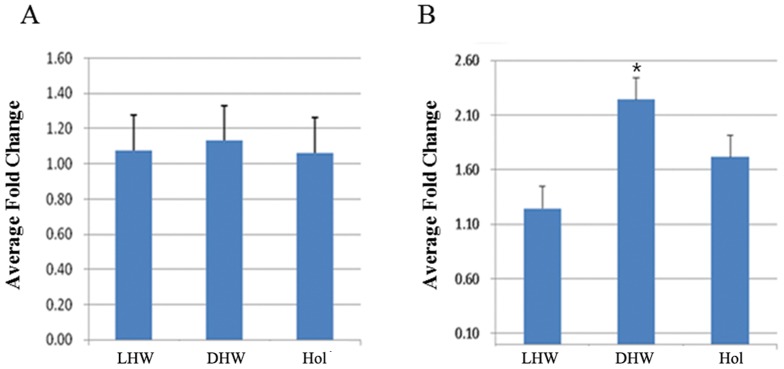
Increased expression of 2 novel genes in dark-muzzle cows. Real-time RT-PCR was performed on RNAs extracted from the muzzle tissue of dark-muzzle HanWoo (DHW), light-muzzle HanWoo (LHW), and Holstein cows (Hol). Expression of the up-regulated gene in dark and light muzzle of HanWoos. The experiments were repeated 3 times, and data are expressed as the mean (±S.D). *(*p*<0.05). A) NM_001076026, B) XN_588439.

During microarray analysis, several genes with strong expression but with unknown function were also noted in dark-muzzle cows. Through gene clustering, we selected 10 novel genes with the most significant expression from dark-muzzle cows and further examined their expression using real-time RT-PCR. Among these, expression of *NM_001076026* and *XM_588439* was higher in cows with a dark-muzzle than in cows with light-muzzle or in the Holstein breed (Fig. 4) The increment was most prominent in the expression of *XM_588439* (*p*<0.05). The difference in the expression of *NM_001076026* was not as pronounced as that of *XM_588439*, but was still statistically significant (*p*<0.05).

**Figure 5:**
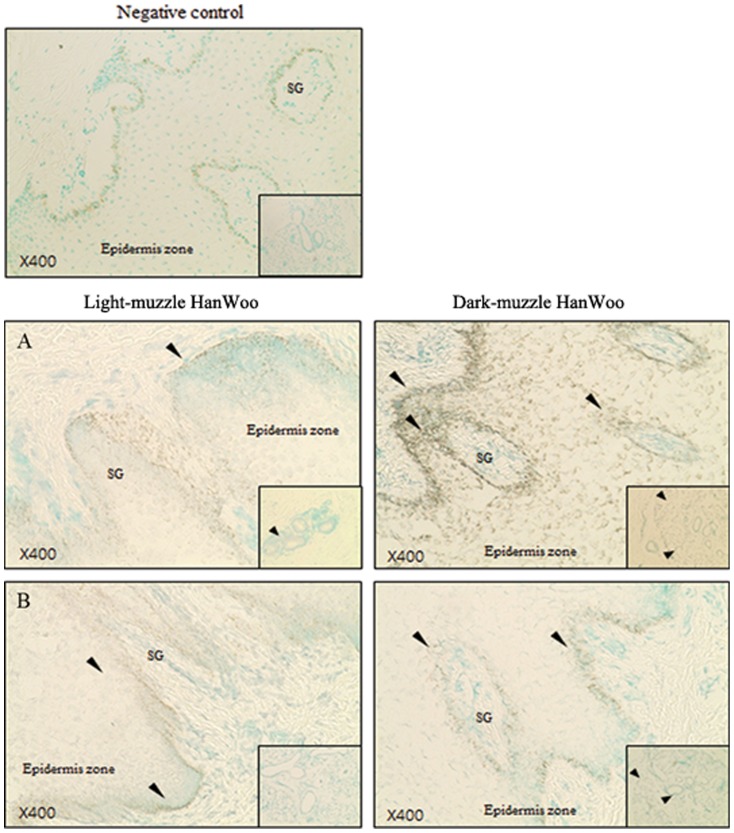
In situ hybridization analysis of highly expressed novel genes in the dark-muzzle HanWoo. The prehybridization solution was used as a negative control. Large figure is the epidermis zone on muzzle. Small figure is the muscle zone on muzzle. Magnification ×400. Arrowheads indicate the regions of strong expression. A) NM-01076026, B) XM-588439, SG: sweat gland.

We examined the tissue distribution of NM_001076026 and XM_588439 transcripts by *in situ* hybridization. NM_001076026 expression was strongest in the skin epithelium of dark-muzzle cows (Fig. 5A). XM_588439 transcripts were also detected in an area similar to NM_001076026, although to a lesser degree (Fig. 5B). Expression of both NM_001076026 and XM_588439 was not obvious in the tissue of light-muzzle cows, indicating that the products of these genes are enriched in the muzzle with black spots.

## Discussion

It has been shown that genes associated with melanogenesis play important roles in the coat color determination of mammals. The synthesis of both pheomelanin and eumelanin is controlled by the interaction among a panoply of genes including TYR and MC1R. Interestingly, a genetic variation in the *MC1R* gene, which codes for the melanocyte-stimulating hormone α (αMSH) receptor, has been shown to be associated with the coat color of cows [13], [14], [15].

Melanogenesis also involves other signaling pathways, in particular, the Wnt and MAPK signaling pathways that affect the synthesis and activity of MITF [16], [17], [18]. Activation of Wnt signaling suppresses β-catenin and GSK-3α, while stimulating LEF in the TCF complexes, which induces the synthesis of MITF [19], [20], [21]. MAPK signaling activates tyrosine metabolism not only by inducing the production of MITF (which in turn activates eumelanin synthesis) but also by controlling the expression of CREB [22], [23], [24].

In contrast, relatively little is known about the genes involved in the determination of nose color. Since the variation in nose color is observed among cows with same coat color, it may employ pathways additional to the ones associated with coat color determination. It appears that some of the genes responsible for determining the coat color also affect pigment deposition in the muzzle of cows. According to a study by Lee et al. (2002), the presence of black spots in the muzzle is associated with the E+ allele of the MC1R locus. Another group also reported an association between the coat color and muzzle pigmentation in Korean brindle cattle [12]. Unfortunately, little is known about the genetic basis of the relationship between muzzle color and melanogenesis. In the present study, we carried out microarray analyses of yellow HanWoo with and without muzzle pigmentation. A total of 5,094 genes showed differences in expression between dark and light muzzle cows, of which 2,820 were up-regulated by more than 2-fold in the muzzle with black pigmentation, while 2,274 were down-regulated. Among these, we identified 12 genes involved in melanogenesis and confirmed their differential expression by real-time RT-PCR.

The identity of some of these genes provides intriguing insight into the possible mechanism through which muzzle pigmentation is regulated. For example, high expression of the AC (Adenylate cyclase 1) family genes, which interact with MC1R may affect the expression of CREB [25], [26], leading to the over-expression of melanogenesis-associated genes such as *PRKACB* and *CAMK2α* [27]. Further, increased expression of genes involved in tyrosine metabolism, such as *TYR*, is likely to affect the synthesis of eumelanin in the dark-muzzle cows [28]. The genetic variation in the MC1R locus may be relayed to the synthesis of MITF via the activation of PKA, which in turn triggers MAPK signaling and TYR expression in dark-muzzle cows [28], [29]. On the other hand, expression of FZD, an upstream regulator of the Wnt signaling pathway [26], was lower in dark-muzzle cows than in light-muzzle cows. In this case, activation of Wnt signaling is likely to inhibit GSK-3β, and, as a result, MAPK signaling is quenched. This will lead to the decreased activity of MITF and TYR synthesis; thus, the relative concentration of pheomelanin will exceed that of eumelanin.

Currently, little is known about how genetic variation in the MC1R locus is relayed to the control of muzzle pigmentation, although many factors are believed to be involved in the regulatory cascade series. In this regard, novel differentially expressed genes from our microarray analyses may provide valuable information. We confirmed higher expression of 2 novel genes, *NM_001076026* and *XM_588439*, in the pigmented muzzle tissue. Overall, *XM_588439* produced a stronger *in situ* signal, but *NM_001076026* transcripts were concentrated in the epithelial layer of the muzzles. Further investigation of these novel genes may help to probe the molecular link between melanogenesis and muzzle color in cows.

To summarize, we performed a microarray-based comparison of the gene expression profiles of muzzle tissue from dark and light muzzle cows. It appears that the activation of MITF downstream of MC1R via MAPK signaling leads to increased synthesis of eumelanin in dark-muzzle cows. In light-muzzle cows, Wnt signaling takes the lead in melanogenesis control, leading to decreased activity of MITF and enrichment of pheomelanin. Identification of novel genes differentially expressed in the dark and light muzzle cows may provide additional information about the molecular mechanism of melanogenesis control, and these can potentially be used as new genetic markers for the determination of coat and muzzle color in cows.

## Conclusions

Using microarray and real-time RT-PCR techniques, we compared the gene expression profiles of muzzle tissues from native Korean cows (HanWoo) with and without black spots in their muzzles. We found an intriguing distinction between dark and light muzzle cows with respect to the expression of genes involved in the MAPK and Wnt signaling pathways. We also noticed increased expression of 2 novel genes in dark-muzzle cows and confirmed the presence of their transcripts in the muzzle epithelium using *in situ* hybridization. Collectively, our results provide new insights into the molecular mechanism of melanogenesis in the muzzle of cows. Genes noted for differential expression in dark and light muzzle cows may serve as new genetic markers for determining muzzle color phenotype.
